# Handy Method for Hypodermy

**Published:** 1873-10

**Authors:** 


### HANDY METHOD FOR HYPODERMY.

A writer in the *British Medical Journal*, Dr. John Crombie, writes :—

"In consequence of the dearness and delicacy of the syringe, the hypodermic application of morphia, although by far the most beneficial when the drug has to be continued for any length of time, has not come to be so extensively used as otherwise it would. I have devised a singular and much less expensive method, by coating a fine silk thread with the required quantity of morphia, which, when introduced in the manner of a seton, by means of a fine needle, with the eye close to the point, enables us to deposit the morphia beneath the skin, by slowly drawing the thread through the opening. This suppository has to be dipped in water immediately before application, and as it is passed through, which must be done slowly, a drop or two let fall on the end still to be passed, so as to moisten the morphia. The process does not cause more pain than injection by the syringe, even when the point of the latter is in condition, as only one-eight of an inch of skin is traversed, and, as it simply requires a needle, does not entail expense, either for purchase or repairs ; and, so I should hope, may be the means of extending the benefit of subcutaneous morphia among the poor, for wh m the syringe is too dear and delicate an instrument."

